# IMPACT OF TRIALS ON CLINICAL PRACTICE: INTERVENTIONS IN SEPTIC SHOCK PATIENTS BETWEEN 2005 AND 2013

**DOI:** 10.1186/2197-425X-3-S1-A430

**Published:** 2015-10-01

**Authors:** A Jones, A Bond, C Whiteley, B Cendreda, M Shankar-Hari

**Affiliations:** Guy's and St Thomas' NHS Foundation Trust, Critical Care Medicine, London, United Kingdom; King's College London, Asthma Allergy and Lung Biology, London, United Kingdom

## Introduction

Evidence based medicine [EBM] at bedside, a key healthcare quality measure, refers to the compendium for delivering optimum clinical care by balancing benefit-harm-costs. EBM involves appraisal, interpretation and implementation with adoption of beneficial interventions and de-adoption of interventions with potential harm.

***Our hypothesis from*** Niven et al [1] where the reversal of intervention effect was not associated with timely de-adoption, is that for a rapid change in clinical practice perceived cost [monetary or clinical harm] attributable to the intervention must be high.

Tight glucose control [TGC] and corticosteroids are examples of nonproprietary and recombinant Activated protein C [rt-APC] an example of proprietary intervention with reversal of effect between publications, from benefit to harm. All three interventions were part of the Surviving Sepsis Campaign [SSC] EBM [2].

## Objectives

We explored the impact of reversal of intervention effect in septic shock trials on the adoption - de-adoption cycle of these three interventions; hypothesis being visible 'cost' influences EBM.

## Methods

Guy's and St. Thomas' NHS Foundation Trust (London, England) is a 1,150-bed, University hospital with closed mixed medical and surgical ICUs and an early adopter of the SSC. Trained data collectors prospectively recorded all ICU admissions with severe sepsis/septic shock (SS) [2005 to 2013] into the SSC database. We report the adoption - de-adoption cycle of the interventions with effect reversal [rt-APC, corticosteroids, TGC] or unchanged (Antibiotics < 3hours; lactate measurement < 6 hours and lung protective ventilation [LPV]) over this period, relative to seminal publications for each intervention in septic shock patients. [2] As an on-going hospital approved audit since inception, informed consent was waived. Data analysis was performed using Stata v13.1 (StataCorp, LP).

## Results

**Figure 1:**
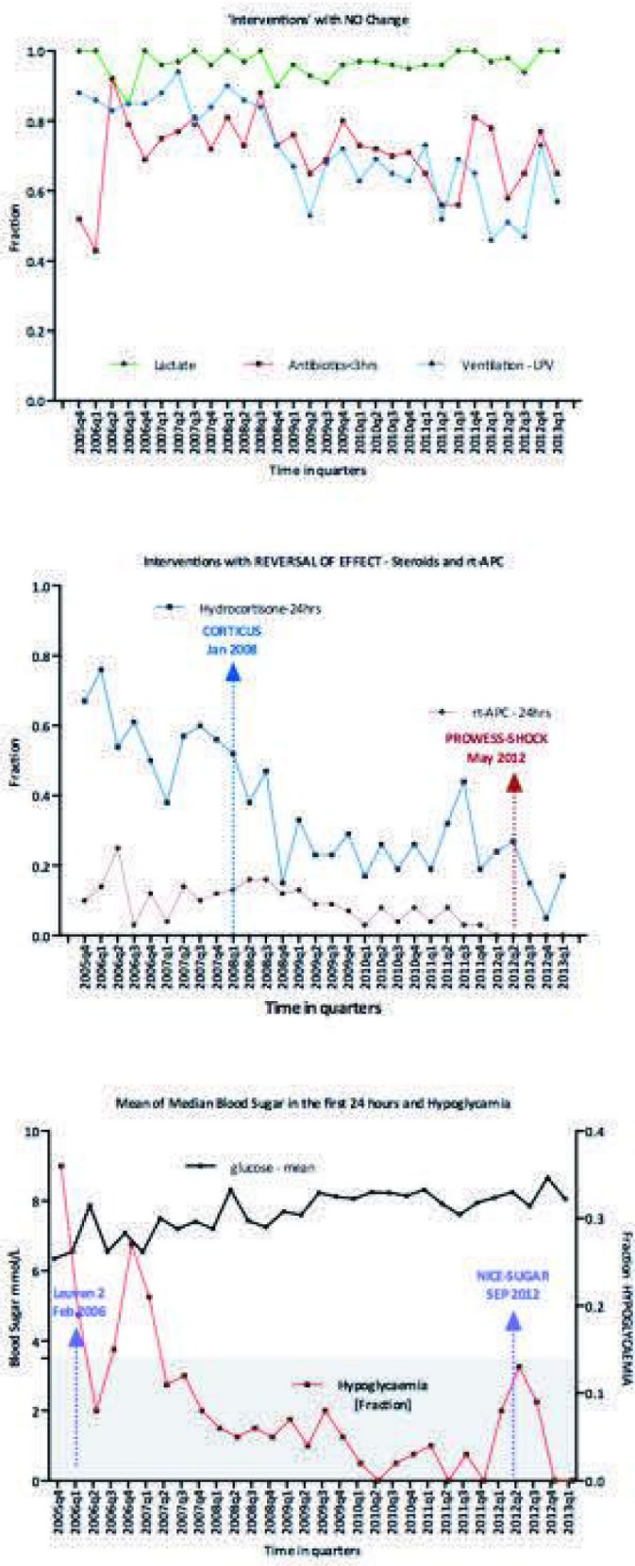
**1a)No vs 1b) Reversal vs 1c) Blood Sugar.**

N = 1,150 septic shock admissions. Compliance with intervention effect unchanged [antibiotics, lactate measurement and LPV] was high [Figure 1a]. Publication of CORTICUS [3]trial reduced steroid use, whereas with the publication of PROWESS-SHOCK [4] study alongside drug withdrawal stopped rt-APC use [Figure 1b]. Between the publications of Leuven-2 [5] and NICE-SUGAR [6] studies, the population average glucose values by quarter increased gradually from 5.7 to 7.6mmol/L, over the study period. This was associated with reduction in hypoglycemia incidence [Figure 1c].

## Conclusions

This descriptive analysis supports our hypothesis. Further analysis will identify key drivers for 'timely' EBM beyond SSC bundle compliance.
